# *Neisseria meningitidis* Group A IgG1 and IgG2 Subclass Immune Response in African Children Aged 12–23 Months Following Meningococcal Vaccination

**DOI:** 10.1093/cid/civ505

**Published:** 2015-11-09

**Authors:** Daniel Holme, Helen Findlow, Samba O. Sow, Olubukola T. Idoko, Marie-Pierre Preziosi, George Carlone, Brian D. Plikaytis, Ray Borrow

**Affiliations:** 1 Vaccine Evaluation Unit, Public Health England, Manchester Royal Infirmary, United Kingdom; 2 Centre pour le Développement des Vaccins, Ministère de la Santé, Bamako, Mali; 3 Medical Research Council Unit, Basse, The Gambia; 4 Meningitis Vaccine Project, PATH, Ferney-Voltaire, France; 5 Meningitis Vaccine Project, Department of Immunization, Vaccines and Biologicals, World Health Organization, Geneva, Switzerland; 6 Centers for Disease Control and Prevention, Atlanta, Georgia

**Keywords:** meningococcal, vaccination, IgG subclass, *Neisseria meningitidis*

## Abstract

***Background.*** A group A meningococcal conjugate vaccine, PsA-TT, was licensed in 2010 and was previously studied in a phase 2 clinical trial to evaluate its safety and immunogenicity in African children 12–23 months of age.

***Methods.*** Subjects received either PsA-TT; meningococcal group A, C, W, Y polysaccharide vaccine (PsACWY); or *Haemophilus influenzae* type b conjugate vaccine (Hib-TT). Forty weeks following primary vaccination, the 3 groups were further randomized to receive either PsA-TT, one-fifth dose of PsACWY, or Hib-TT. Group A–specific immunoglobulin G (IgG) subclass response was characterized using an enzyme-linked immunosorbent assay.

***Results.*** The predominant IgG subclass response, regardless of vaccine, was IgG1. One month following primary vaccination, the geometric mean concentrations (GMCs) of IgG1 and IgG2 in the PsA-TT group were 21.73 µg/mL and 6.27 µg/mL, whereas in the PsACWY group the mean GMCs were 2.01 µg/mL and 0.97 µg/mL, respectively (*P* < .0001). Group A–specific IgG1 and IgG2 GMCs remained greater in the PsA-TT group than in the PsACWY group 40 weeks following primary vaccination (*P* < .0001). One week following revaccination, those given 2 doses of PsA-TT had the greatest IgG1 and IgG2 GMCs of 125.23 µg/mL and 36.12 µg/mL, respectively (*P* = .0008), and demonstrated a significant increase in IgG1:IgG2 mean ratio, indicative of the T-cell–dependent response associated with conjugate vaccines.

***Conclusions.*** Vaccination of African children aged 12–24 months with either PsA-TT or PsACWY elicited a predominantly IgG1 response. The IgG1:IgG2 mean ratio decreased following successive vaccination with PsACWY, indicating a shift toward IgG2, suggestive of the T-cell–independent immune response commonly associated with polysaccharide antigens.

***Clinical Trials Registration.*** SRCTN78147026.

Sub-Saharan Africa has experienced epidemic cycles of *Neisseria meningitidis* group A (MenA) disease approximately every 5–10 years, with high disease incidence. One of the worst epidemics occurred in 1996, with greater than 250 000 recorded cases and 25 000 deaths [1]. The specific area of sub-Saharan Africa in which epidemics of MenA disease are frequent is termed the “meningitis belt” and was first described by Lapeyssonnie in 1963 [2] as spanning from Senegal in the west to Ethiopia in the east.

Polysaccharide vaccines against MenA have been used in response to African outbreaks. These vaccines, however, are poorly immunogenic in children <2 years of age due to low numbers of mature B cells [3], whereas polysaccharide protein conjugate vaccines are immunogenic in infants and induce immune memory [4, 5].

In 2001, the Meningitis Vaccine Project, a partnership between PATH and the World Health Organization, secured funding for the development, testing, licensure, and introduction of an effective meningococcal MenA conjugate vaccine designed specifically for use in Africa at a low cost [6]. A phase 1 clinical study of a MenA conjugate vaccine, PsA-TT (Serum Institute of India, Ltd), was successfully carried out in adult volunteers in India [7], and phase 2 and 2/3 clinical studies were performed in 1- to 29-year-olds in Africa and India [8]. The studies demonstrated that a single dose of PsA-TT was safe in children and induced a superior immune response and immune memory compared with the polysaccharide vaccine, as demonstrated by serum bactericidal antibody (SBA) assay and anti-group A–specific immunoglobulin G (IgG) enzyme-linked immunosorbent assay (ELISA) [8].

Polysaccharide vaccines elicit a T-cell–independent response which, in adults, produces increased concentrations of IgG2 relative to IgG1 [9]. In infants, the T-cell–independent response is poor, so IgG2 production is thought to be negligible. The inability of young children to produce a significant IgG2 response to polysaccharides can be overcome by first priming with a conjugate vaccine to the same antigen [10]. Like polysaccharide vaccines, conjugate vaccines predominantly induce IgG1 in infants [11]. We report here on the IgG1 and IgG2 antibody subclass response in African children following vaccination with PsA-TT or PsACWY.

## MATERIALS AND METHODS

### Study Group

The full details of this study group have been reported elsewhere [8]. In brief, healthy children (12–23 months old) who were fully vaccinated according to the local Expanded Programme on Immunization (EPI) schedule were recruited from 2 urban quarters in Bamako, Mali, and in Basse, which is in the Upper River Region of The Gambia. The clinical trial is registered (number SRCTN78147026) at www.controlled-trials.com.

### Vaccines and Vaccination

PsA-TT vaccine is available as a lyophilized 10-dose vial to be reconstituted with a 5-mL diluent ampoule. A single 0.5-mL dose of the reconstituted PsA-TT vaccine contained 10 µg of purified MenA polysaccharide conjugated to 10–33 µg of tetanus toxoid (TT) carrier protein with aluminum phosphate as an adjuvant, tris (hydroxymethyl) aminoethane as a buffer, 0.9% sodium chloride, 0.01% thimerosal preservative, and sterile water for injection (investigational vaccine; MenAfriVac, PsA-TT, Serum Institute of India Ltd, Pune). A single 0.5-mL dose of PsACWY vaccine contained 50 µg of each meningococcal ACWY polysaccharide (Mencevax, ACWY, GlaxoSmithKline [GSK], Belgium). A single dose of the reconstituted Hib-TT vaccine contained 10 µg of purified Hib–polyribosylribitol phosphate conjugated to 20–40 µg of TT (Hiberix, GSK). All initial doses of vaccine were administered intramuscularly in the right thigh. For revaccination, PsA-TT and Hib vaccine were administered intramuscularly in the right deltoid, whereas the one-fifth dose of PsACWY was administered subcutaneously in the right deltoid.

Subjects were randomized in a 1:1:1 mean ratio to 1 of 3 groups to receive either primary vaccination of PsA-TT, PsACWY reference, or control Hib-TT vaccine. Forty weeks following primary vaccination, subjects in each primary vaccination group were further randomized in a 1:1:1 mean ratio to receive either PsA-TT, Hib-TT, or one-fifth of a full dose of PsACWY, resulting in 9 vaccine groups at revaccination.

### Serologic Sample Time Points

Blood samples were collected prior to the primary injection (week 0), at 28 days (week 4), prior to revaccination (40 weeks after primary vaccination), 7 days following revaccination (week 41), and 28 days following revaccination (week 44).

### Meningococcal Group A–Specific IgG1 and IgG2 ELISA

Samples collected at each time point were analyzed in the group A–specific IgG1 and IgG2 ELISA as previously described by Joseph et al [12]. In brief, microtiter plates were coated with a mixture of MenA polysaccharide (National Institute for Biological Standards and Control [NIBSC], Potters Bar, UK) and methylated human serum albumin (NIBSC) in phosphate-buffered saline. Following nonspecific protein binding blocking, a reference (CDC1992, NIBSC), a quality control, and unknown sera were added in duplicate and eight 2-fold dilutions made directly in the plate. Following overnight incubation, plates were incubated sequentially with murine monoclonal antibodies to human IgG1 (clone HP6069, Life Technologies Ltd, Paisley, UK) or IgG2 (clone HP6002, Life Technologies Ltd) for 3 hours at room temperature, followed by rabbit antimouse, horseradish peroxidase–conjugated secondary antibody for 2.5 hours at room temperature. Plates were then developed with chromogenic substrate tetramethylbenzidine dihydrochloride monohydrate (Sigma-Aldrich, Dorset, UK). The optical density of each well was then read at 450 nm. MenA-specific IgG and IgG subclass concentrations were calculated with a 4-parameter, logistic curve model in the SOFTmax PRO (Molecular Devices, Wokingham, UK) data analysis software program. Assays were conducted by the Public Health England Vaccine Evaluation Unit (Manchester, UK).

### Statistical Analysis

Anti-MenA–specific IgG1 and IgG2 GMCs and 95% confidence intervals (CIs) were calculated. The GMCs were adjusted for the concentration at week 0, age, sex, and site. These estimates took into account the longitudinal nature of the data, defining all observations from each individual as a separate cluster. As a result, within-person or within-cluster sources of variation were accounted for in the analysis.

Concentrations and ratios of concentrations (IgG1:IgG2) were log-transformed prior to analysis. Data were analyzed using a longitudinal mixed-model analysis of variance, and linear contrasts were calculated between groups at each visit and between visits. Results were back-calculated using anti-logs to generate GMCs and mean ratios of concentrations with 95% CIs. Two-tailed *P* values were derived to test whether the mean IgG1:IgG2 mean ratios differed from 1.0.

## RESULTS

Of the samples available for analysis (n = 2881), results were achieved for 1747 samples for IgG1 and 2023 samples for IgG2. Samples collected from those primed with Hib-TT at weeks 0 and 4 were not analyzed for their MenA-specific IgG1 and IgG2 responses because this group was not a priority for this analysis and concentrations of MenA-specific IgG were low [8].

Tables 1 and 2 show the IgG1 and IgG2 GMCs for all time points. Table 3 shows the mean ratios of IgG1:IgG2 at each time point.

**Table 1.** Group A Immunoglobulin G1 Geometric Mean Concentration Summary for Randomized Vaccine Groups, Weeks 0–44, With a Primary Vaccine Administered at Week 0 and Revaccination at Week 40

Primary Vaccine	Group A–Specific IgG1 GMCs (95% CI), µg/mL					
	Week 0	Week 4	Week 40	Revaccination	Week 41	Week 44
PsA-TT^a^	0.46 (.40–.52)	21.73 (19.14–24.67)	1.29 (1.12–1.48)	PsA-TT	125.23 (93.46–170.50)	69.13 (51.60–92.61)
				PsACWY	30.72 (22.78–41.43)	19.20 (14.72–25.04)
				Hib-TT	2.12 (1.25–3.34)	1.61 (1.09–2.37)
PsACWY^b^	0.62 (.54–.70)	2.01 (1.74–2.33)	0.82 (.71–.94)	PsA-TT	36.26 (25.34–51.88)	27.03 (19.23–38.00)
				PsACWY	4.08 (3.09–5.38)	2.98 (2.26–3.94)
				Hib-TT	0.77 (.61–.97)	0.73 (.57–.93)
Hib-TT^c^	NA	NA	0.44 (.36–.53)	PsA-TT	17.99 (11.62–27.86)	20.47 (13.78–30.41)
				PsACWY	4.38 (2.42–7.93)	1.88 (1.26–2.80)
				Hib-TT	0.55 (.33–.89)	0.38 (.27–.53)

Abbreviations: CI, confidence interval; GMC, geometric mean concentration; IgG1, immunoglobulin G1; NA, not applicable.

^a^ Group A meningococcal conjugate vaccine.

^b^ Meningococcal group A, C, W, Y polysaccharide vaccine.

^c^ *Haemophilus influenzae* type b conjugate vaccine.

**Table 2.** Group A Immunoglobulin G2 Geometric Mean Concentrations for Randomized Vaccine Groups, Weeks 0–44, With a Primary Vaccine Administered at Week 0 and Revaccination at Week 40

Primary Vaccine	Group A–Specific IgG2 GMCs (95% CI), µg/mL					
	Week 0	Week 4	Week 40	Revaccination	Week 41	Week 44
PsA-TT^a^	0.10 (.08–.12)	6.27 (5.25–7.50)	0.76 (.63–.91)	PsA-TT	36.12 (25.22–51.75)	33.27 (23.02–48.06)
				PsACWY	13.27 (10.26–17.18)	15.24 (11.78–19.72)
				Hib-TT	0.70 (.48–1.00)	0.71 (.44–1.15)
PsACWY^b^	0.14 (.11–.16)	0.97 (.80–1.18)	0.36 (.29–.44)	PsA-TT	15.97 (11.70–21.80)	26.82 (19.65–36.61)
				PsACWY	2.04 (1.39–2.98)	2.25 (1.53–3.31)
				Hib-TT	0.32 (.24–.42)	0.25 (.17–.37)
Hib-TT^c^	NA	NA	0.14 (.12–.17)	PsA-TT	3.46 (2.06–5.80)	7.83 (3.72–16.47)
				PsACWY	0.52 (.29–.94)	1.15 (.60–2.18)
				Hib-TT	0.17 (.13–.23)	0.14 (.10–.20)

Abbreviations: CI, confidence interval; GMC, geometric mean concentration; IgG2, immunoglobulin G2; NA, not applicable.

^a^ Group A meningococcal conjugate vaccine.

^b^ Meningococcal group A, C, W, Y polysaccharide vaccine.

^c^ *Haemophilus influenzae* type b conjugate vaccine.

**Table 3.** Group A Immunoglobulin G1: Immunoglobulin G2 Mean Ratios for Randomized Vaccine Groups, Weeks 0–44, With a Primary Vaccine Administered at Week 0 and Revaccination at Week 40

Primary Vaccine	Group A–Specific IgG1:IgG2 Mean Ratios (95% CI)					
	Week 0	Week 4	Week 40	Revaccination	Week 41	Week 44
PsA-TT^a^	4.69 (4.10–5.36)	3.51 (3.06–4.02)	1.65 (1.42–1.92)	PsA-TT	3.74 (2.91–4.79)	1.94 (1.52–2.48)
				PsACWY	2.39 (1.96–2.92)	1.27 (1.06–1.52)
				Hib-TT	2.26 (1.27–4.04)	1.47 (.89–2.42)
PsACWY^b^	4.74 (4.03–5.58)	1.72 (1.43–2.07)	2.26 (1.90–2.68)	PsA-TT	2.43 (1.84–3.21)	1.25 (.96–1.62)
				PsACWY	1.87 (1.45–2.42)	1.28 (.98–1.66)
				Hib-TT	1.77 (1.18–2.65)	1.91 (1.02–3.60)
Hib-TT^c^	NA	NA	2.84 (2.15–3.76)	PsA-TT	3.77 (2.68–5.30)	3.17 (2.10–4.77)
				PsACWY	2.02 (.86–4.75)	1.83 (1.04–3.20)
				Hib-TT	2.83 (1.31–6.12)	3.82 (2.29–6.37)

Abbreviations: CI, confidence interval; IgG1, immunoglobulin G1; IgG2, immunoglobulin G2; NA, not applicable.

^a^ Group A meningococcal conjugate vaccine.

^b^ Meningococcal group A, C, W, Y polysaccharide vaccine.

^c^ *Haemophilus influenzae* type b conjugate vaccine.

### Primary Vaccination

Before vaccination (week 0), IgG1 was the predominant antibody subclass. The IgG1 GMCs for the PsA-TT and PsACWY groups were 0.46 µg/mL (95% CI, .40–.52) and 0.62 µg/mL (95% CI, .54–.70), respectively (Table 1). The IgG1 GMC of the PsACWY group was greater than that of the PsA-TT group (*P* = .001), but this difference only equated to 0.16 µg/mL. The MenA-specific IgG2 GMCs for the PsA-TT and PsACWY groups were 0.10 µg/mL (95% CI, .08–.12) and 0.14 µg/mL (95% CI,.11–.16), respectively (Table 2).

One month following primary vaccination (week 4) with either PsA-TT or PsACWY, both IgG1 and IgG2 GMCs had increased significantly from baseline (*P* < .0001), with the PsA-TT group having significantly greater IgG1 and IgG2 GMCs (*P* < .0001) (Table 1). IgG1 continued to predominate, with IgG1:IgG2 mean ratios of 3.51 and 1.72 for the PsA-TT and PsACWY groups, respectively. The difference between the groups was significant (*P* < .0001) (Table 3).

### Revaccination

Prior to revaccination (week 40), the IgG1 and IgG2 GMCs remained significantly greater in the PsA-TT group than in the PsACWY group, which in turn had greater GMCs than the Hib-TT group (*P* < .0001; Table 1). A shift in distribution toward IgG2 was apparent in the PsA-TT group, as indicated by the decrease in IgG1:IgG2 mean ratio from week 4 to week 40 (*P* < .0001; Table 3). The GMCs of both antibody subclasses in the Hib-TT group were similar to those of the prevaccination groups.

One week following revaccination (week 41), the group given 2 doses of PsA-TT had the highest IgG1 and IgG2 GMCs, which were higher than those in all other vaccine groups (*P* between .0008 and <.0001). There was a significant shift in distribution toward IgG1 in this group, as indicated by the increase in IgG1:IgG2 mean ratio (*P* < .0001). Groups that had been primed with the PsA-TT had higher MenA IgG1 and IgG2 GMCs than did groups primed with PsACWY or Hib-TT if receiving the same vaccine at 40 weeks (*P* < .0010). The same trend was seen in those revaccinated with PsA-TT compared with those revaccinated with PsACWY or Hib-TT after receiving the same priming vaccine (*P* < .0001). Groups given Hib-TT at 40 weeks did not show any significant difference in IgG1 and IgG2 GMCs 1 week after revaccination, with the exception of an increase in IgG1 in the group primed with PsA-TT (*P* = .0156).

One month following revaccination (week 44), those who received 2 doses of PsA-TT had an IgG1 GMC that was still significantly greater than that of all other groups (*P* < .0001). However, this group showed a reduction in IgG1 GMC from week 41 to week 44 (*P* = .0035). The IgG2 GMC in this group also decreased from week 41 to week 44, although not significantly (*P* = .7258). IgG1:IgG2 mean ratios significantly decreased from week 41 to week 44 in those who received 2 doses of PsA-TT, PsA-TT/PsACWY, and PsACWY/PsA-TT (*P* = .0003). This was attributed to either a reduction in IgG1 GMC (2 doses of PsA-TT and PsA-TT/PsACWY) or an increase in IgG2 GMCs (*P* = .0080) (PsACWY/PsA-TT).

Primary and secondary vaccine responses were contrasted by comparing the 4 weeks following primary vaccination (week 4) with the 4 weeks following revaccination (week 44). For IgG1 and IgG2, the secondary vaccine response in the group receiving 2 doses of PsA-TT was 3- and 5-fold greater, respectively, than the primary response. Challenge with PsACWY following PsA-TT vaccination resulted in an improved IgG2 secondary response (*P* < .0001) but not IgG1. Those primed with PsACWY and boosted with PsA-TT had an IgG1 and IgG2 secondary response that was 13-fold and 27-fold greater, respectively, than the primary response.

## DISCUSSION

This study demonstrated that prior to vaccination, baseline levels of IgG1 were significantly greater than those of IgG2, consistent with the current theory that IgG2 production occurs later in ontogeny [13]. MenA-specific IgG1 and IgG2 GMCs increased significantly 4 weeks following vaccination with PsA-TT or PsACWY, with IgG1 predominating. Significantly higher IgG1 and IgG2 GMCs were observed following vaccination with PsA-TT than with PsACWY, but the response was not exclusively IgG1 in either group. Following either PsA-TT or PsACWY vaccination, the IgG1:IgG2 mean ratios indicate a shift toward IgG2, and this was significantly greater after PsACWY vaccination, indicating an early T-cell–independent response that is more commonly associated with adults and children >2 years of age [3]. The mean IgG1:IgG2 ratio, 3.51, observed in this study following PsA-TT vaccination was comparable to that observed previously in Gambian infants following meningococcal group A/C conjugate vaccine (MACC) (3.79) [14].

From week 4 to week 40, the MenA-specific IgG1 and IgG2 GMCs significantly decreased for those receiving PsA-TT and PsACWY. A relatively equal wane of IgG1 and IgG2 was seen in the PsACWY group, resulting in an unchanged mean ratio, whereas the IgG1 wane of the PsA-TT group was twice that of IgG2, resulting in a significant reduction in IgG1:IgG2 mean ratio. The persistence of antibody following one dose of vaccine was not anticipated to be long term following the observation of declining effectiveness 1 year following even multiple doses of meningococcal serogroup C vaccines administered in infancy [15]. The relatively large fall in IgG1 GMCs from week 4 to week 40 in the PsA-TT primed group was expected because the half-life of IgG1 and IgG2 is approximately 21 days [16], and both naturally decrease to levels similar to those at baseline. At week 40, the effect of the 3 different vaccines could still be detected, with significantly different IgG1 and IgG2 GMCs. This was also true of the MenA-specific IgG GMCs reported by Sow et al [8].

Following revaccination, significant increases in both MenA-specific IgG1 and IgG2 GMCs were observed in all vaccine cohorts, with the exception of those revaccinated with Hib-TT. Significantly greater IgG1 and IgG2 GMCs were observed in those who received 2 doses of PsA-TT, mirroring the IgG GMCs reported by Sow et al [8] and consistent with previous studies demonstrating the superiority of multiple doses of conjugate vaccines over polysaccharide vaccines in young children [17].

Revaccination with either PsA-TT or PsACWY following PsA-TT priming changed the distribution of subclasses toward an IgG1 response, consistent with previous findings of an IgG1 predominance following MACC vaccination in infants [14] and meningococcal quadrivalent conjugate vaccination in adults [18]. Children primed with PsACWY and revaccinated with PsACWY showed a shift in distribution toward IgG2. Those in the Hib-TT/PsA-TT group had the greatest IgG1:IgG2 mean ratio at this time point, but statistical significance could not be shown because of the spread of the 95% CIs. This was also true of the CIs for IgG1 and IgG2 GMCs in this group. In part, the lack of statistical significance may be associated with the slightly lower number of subjects achieving a result in this group, although it also may be evidence of immune interference such as carrier-induced epitopic suppression as a result of preexisting immunity to the common TT carrier protein [19].

At week 40, there appeared to be a marked difference in the IgG1 and IgG2 responses. Holistically, 8 of 9 vaccine groups had a reduction in IgG1 GMC from week 41 to week 44, whereas the IgG2 GMC increased in 6 of 9 groups. During the same study time period, Sow et al [8] reported a decline in rabbit complement SBA (rSBA) geometric mean titers (GMTs) in the groups that received 2 doses of PsA-TT, PsA-TT/PsACWY, and PsACWY/PsA-TT, all of which had shown either a significant decrease in IgG1 or increase in IgG2. The reduction in rSBA GMTs was thought to be attributable to the early rapid expansion of antibody-secreting cells resulting in increased antibody production (week 41), followed by a contraction or downregulation of antibody-secreting cells as antigen became less available (week 44). Similar findings have been reported previously and have been attributed to early-onset high-avidity antibodies [20] or a decrease in plasma and memory B cells [21]. Those who received 2 doses of PsA-TT demonstrated a significant reduction in MenA-specific IgG over the same period, further supporting the theory that this may be attributed to downregulation of antibody secreting cells.

Those who received PsACWY followed by PsA-TT did not demonstrate a significant decrease in IgG1 or IgG GMCs but did show an increase in IgG2 GMCs. The IgG1:IgG2 mean ratios showed a significant decrease in all 3 groups over this time period, and the reduction in rSBA GMTs may in fact be due to the greater proportion of lower-avidity IgG2. Previous studies have shown that persistence of IgG2 and a reduction in IgG1:IgG2 mean ratio can correlate to decreased IgG avidity [22]. Alternatively, the reduction in rSBA may be a result of the increased proportion of IgG2 that binds complement less effectively than IgG1 [23].

The decrease in IgG1 GMCs following revaccination from week 41 to week 44 was unexpected because the peak and then plateau of antibody response following meningococcal C conjugate (MCC) vaccination has been demonstrated to be 2–4 weeks for an MCC vaccine [24] and 2–8 weeks for a polysaccharide [25]. In those who received 2 doses of PsA-TT, the peak at week 41 and decline to week 44 following the second dose was not seen in the antitetanus IgG GMCs, with GMCs of 9.93 IU/mL (95% CI, 7.92–12.44) and 10.21 IU/mL (95% CI, 8.13–12.85) at weeks 41 and 44, respectively.

The PsA-TT/PsACWY group demonstrated a significantly increased response with regard to IgG2 GMC following revaccination compared to primary vaccination. There was a significant decrease in the IgG1:IgG2 ratio from primary vaccination to before the 1-month booster. This is consistent with the findings of Findlow [14], who demonstrated a significant increase in group A IgG2 and decrease in IgG1:IgG2 in infants after administration of a meningococcal A/C polysaccharide booster following MACC priming. Similar to what is reported here, the MenA IgG1 GMC remained at the same level 1 month following revaccination, as it did 1 month following primary vaccination [14]. An increased immune response to polysaccharide vaccines following priming with a conjugate vaccine is evidence of the stimulation of previously induced memory B cells [26]. A large proportion of the response was IgG1, which is indicative of the T-cell–dependent response [11]. The large increase in IgG2 suggests that a T-cell–independent type response was also induced, indicating that the response to group A is mixed [27].

Successive vaccination with polysaccharide vaccines may induce hyporesponsiveness in children [28, 29] and adults [30, 31]. Although the 2-dose PsACWY group did not demonstrate hyporesponsiveness to MenA, the same study population did show hyporesponsiveness for groups C and W using rSBA [32]. The responses of the IgG1 and IgG2 GMCs 1 month following primary vaccination to 1 month following revaccination indicated small, yet significantly greater IgG1 (*P* = .0105) and IgG2 (*P* < .0001) booster response compared with that of the primary vaccination, consistent with the response previously observed following successive doses of group A meningococcal polysaccharide in infants aged 7–12 months [33].

The trend in the PsACWY/PsACWY group of a significant reduction in rSBA titer between 1 week and 1 month after booster vaccination was not seen in the MenA-specific IgG GMCs. The MenA IgG1 and IgG2 GMCs presented here demonstrated a high degree of correlation (*r* = 0.8485–0.9432 [data not shown]) to the IgG concentrations described by Sow et al [8]. This group had shown a shift in subclass distribution toward IgG2 after each successive PsACWY vaccination as is consistent with a T-cell–independent response [34].

The data generated here, along with the data previously reported by Sow et al [8], were compared for the PsACWY/PsACWY and PsACWY/PsA-TT groups. The comparison indicated that hyporesponsiveness was partially overcome by revaccinating with PsA-TT, resulting in rSBA titers, group A IgG, IgG1, and IgG2 GMCs that were 9 times that of the PsACWY/PsACWY group. The PsACWY/PsA-TT group was not significantly different from the PsA-TT/PsA-TT group in terms of rSBA, IgG, and IgG2 but did have a significantly lower IgG1 GMC (*P* < .0001), further demonstrating the prolonged effect of polysaccharide vaccination following conjugate vaccination. MCC vaccines have been shown previously to partially overcome the effect of hyporesponsiveness in children [28, 31]; this has now been demonstrated with group A conjugates.

Vaccination of African children aged 12–23 months with either PsA-TT or PsACWY elicited a predominantly IgG1 response. The distribution of antibody subclasses was shown to shift toward IgG2 following vaccination with PsACWY, typical of T-cell–independent type 2 antigens. Priming with the PsA-TT and revaccination with PsACWY produced an increased MenA IgG2 response but not an increased IgG1 response. This may be attributed to the T-cell–independent nature of the PsACWY, which may activate not only the previously stimulated memory B cells but also other T-cell–independent immune cells such as marginal zone B cells [35]. A significantly increased immune response in those primed and boosted with the PsA-TT was observed for both IgG1 and IgG2. The decrease previously reported in rSBA and group A–specific IgG [8] between 1 week and 1 month following revaccination in some vaccine groups may be attributed to the rapid expansion and subsequent downregulation of antibody secretion due to the reduced availability of antigen. This was mirrored by either a significant decrease in IgG1 and or IgG1:IgG2 mean ratio between the 2 time points. A greater proportion of IgG2 that may be of lower avidity and/or that binds complement less effectively may also offer an explanation for the reduced activity. The cause of the rapid decline in IgG1 remains a topic for future studies.
